# Whole‐brain monosynaptic inputs to lateral periaqueductal gray glutamatergic neurons in mice

**DOI:** 10.1111/cns.14338

**Published:** 2023-07-09

**Authors:** Wei‐Xiang Ma, Lei Li, Ling‐Xi Kong, Hui Zhang, Ping‐Chuan Yuan, Zhi‐Li Huang, Yi‐Qun Wang

**Affiliations:** ^1^ Department of Pharmacology, School of Basic Medical Sciences, State Key Laboratory of Medical Neurobiology and MOE Frontiers Center for Brain Science, and Institutes of Brain Science Fudan University Shanghai China; ^2^ Anhui Provincial Engineering Research Center for Polysaccharide Drugs, Provincial Engineering Laboratory for Screening and Re‐evaluation of Active Compounds of Herbal Medicines in Southern Anhui, School of Pharmacy Wannan Medical College Wuhu China

**Keywords:** glutamatergic neurons, lateral periaqueductal gray, monosynaptic inputs, rabies virus

## Abstract

**Objective:**

The lateral periaqueductal gray (LPAG), which mainly contains glutamatergic neurons, plays an important role in social responses, pain, and offensive and defensive behaviors. Currently, the whole‐brain monosynaptic inputs to LPAG glutamatergic neurons are unknown. This study aims to explore the structural framework of the underlying neural mechanisms of LPAG glutamatergic neurons.

**Methods:**

This study used retrograde tracing systems based on the rabies virus, Cre‐LoxP technology, and immunofluorescence analysis.

**Results:**

We found that 59 nuclei projected monosynaptic inputs to the LPAG glutamatergic neurons. In addition, seven hypothalamic nuclei, namely the lateral hypothalamic area (LH), lateral preoptic area (LPO), substantia innominata (SI), medial preoptic area, ventral pallidum, posterior hypothalamic area, and lateral globus pallidus, projected most densely to the LPAG glutamatergic neurons. Notably, we discovered through further immunofluorescence analysis that the inputs to the LPAG glutamatergic neurons were colocalized with several markers related to important neurological functions associated with physiological behaviors.

**Conclusion:**

The LPAG glutamatergic neurons received dense projections from the hypothalamus, especially nuclei such as LH, LPO, and SI. The input neurons were colocalized with several markers of physiological behaviors, which show the pivotal role of glutamatergic neurons in the physiological behaviors regulation by LPAG.

## Graphical abstract

RV‐based retrograde tracers allow us to identify neuronal presynaptic connections. we identified that seven brain structures, namely the cortex, thalamus, hypothalamus, superior colliculus, midbrain, pons, and medulla, project monosynaptic inputs to the LPAG glutamatergic neurons, in which the hypothalamus project most densely.

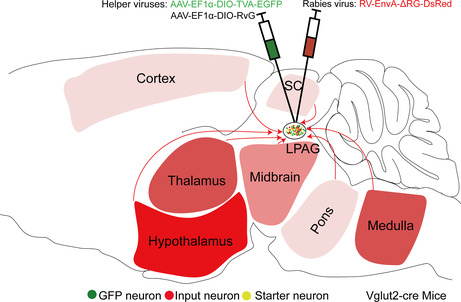

## 1 INTRODUCTION

The periaqueductal gray (PAG) is located in the midbrain and regulates multiple functions, including defensive,^1^ social,^2^ antinociceptive,^3^ itch‐scratching,^4^ and emotional behaviors.^5^ From the dorsal to ventral directions, the region is divided into four function‐specific columns, namely the dorsomedial, dorsolateral, lateral (LPAG), and ventrolateral (VLPAG) columns.^5^, ^6^

As the lateral part of the PAG, the LPAG comprises a heterogeneous nucleus in terms of neurotransmitter types and mainly contains glutamatergic and GABAergic neurons. The LPAG is involved in social,^7^ pain,^8^ fear,^9^ offensive, and defensive behaviors.^10^, ^11^, ^12^ There may be differences in the regulatory functions of different types of neurons in the LPAG. For example, the LPAG^Vgat^ neurons support prey search, chase, and attack behaviors; however, LPAG^Vglut2^ neurons are involved in supporting the attack behavior only.^12^ In addition, LPAG is also involved in sleep–wake regulation. A previous study reported that LPAG neurotensinergic neurons promote non‐rapid eye movement (NREM) sleep.^13^ Although the LPAG can regulate many physiological behaviors, it is unclear which specific excitatory or inhibitory signals directly control LPAG. Therefore, identification of the whole‐brain inputs to LPAG can help to better understand the regulation of LPAG activity in different behavioral processes.

Rabies virus (RV)‐based retrograde tracers allow us to identify neuronal presynaptic connections^14^, ^15^ and can be used to overcome the limitations of previous studies. In this study, we identified whole‐brain monosynaptic inputs to LPAG glutamatergic neurons using transgenic mice that express Cre recombinase in glutamatergic neurons and combined a genetically modified RV with Cre‐LoxP technology. Our results revealed that 59 afferent brain nuclei, and several nuclei with the highest input density (such as LH, LPO, and VP), are involved in the regulation of physiological behaviors. In conclusion, our findings provided substantial evidence for the structural framework of LPAG glutamatergic neurons and may guide neuronal pathway studies of glutamatergic neuron function in the LPAG.

## 2 MATERIALS AND METHODS

### 2.1 Animals

Adult transgenic mice (10–12‐week‐old, 25–28 g) with Cre recombinase expression in glutamatergic neurons (Vglut2‐Cre mice) of the C57BL/6J strain and wild‐type littermates without Cre expression were used for retrograde tracing experiments. The mice were housed under an automatically controlled 12/12‐h light/dark cycle (lights on at 7 a.m.; intensity of 100 lux)^16^ at a constant temperature of 22 ± 0.5°C and relative humidity of 60% ± 2%. The mice were provided free access to food and water. All animal experiments in this study were approved by Laboratory Animal Model Department, Shanghai Public Health Clinical Center, Fudan University (Permit No. 2023‐A008‐01).

### 2.2 Virus

BrainVTA (Wuhan, China) was responsible for packaging the viral vectors. The titers of AAV‐EF1α‐DIO‐TVA‐EGFP, AAV‐EF1α‐DIO‐RvG, and an EnvA‐pseudotyped glycoprotein (RG)‐deleted and DsRed‐expressing RV (RV‐EnvA‐ΔRG‐DsRed) were 5 × 10^12^, 5 × 10^12^, and 2 × 10^8^ genomic copies/mL, respectively.

### 2.3 Surgery and viral injections

The surgical procedures were performed according to previous studies.^17^ Briefly, the mice were anesthetized by intraperitoneal injection of chloral hydrate (350 mg/kg) and were fixed in a stereotaxic apparatus. Then, the mice skulls were exposed and a small hole was drilled. The viral vectors were microinjected into the unilateral LPAG via a micropipette. The coordinate of LPAG was as follows: −4.10 mm from bregma, 0.35 mm lateral from midline, and 2.30 mm vertical from the pial surface. For retrograde tracing, helper viruses (AAV‐EF1α‐DIO‐TVA‐EGFP and AAV‐EF1α‐DIO‐RvG were mixed at a 1:1 ratio; 100 nL) were injected into the LAPG and left for 15 min to allow diffusion away from the injection site. Three weeks later, RV‐EnvA‐ΔRG‐DsRed (50 nL) was microinjected into the same location (*n* = 4 mice). At week 4, the mice were perfused.

### 2.4 Histology and immunostaining

One week after injection of the RV, the mice were deeply anesthetized and perfused with phosphate‐buffered saline (PBS), followed by 4% paraformaldehyde in 0.1 M phosphate buffer (PB, pH 7.4). Following perfusion, the brains were removed and post‐fixed in 4% PFA for 4–6 h at 4°C and then cryoprotected in 10%, 20%, and 30% sucrose at 4°C until they sank. Coronal brain sections (30‐μm‐thick) were cut on a freezing cryostat (CM1950; Leica, Wetzlar, Germany) into four series and were collected in 0.1 M PBS (pH 7.4).

For immunofluorescence, the brain sections were rinsed three times with 0.01 M PBS containing 0.3% Triton X‐100 (PBST) and then incubated overnight at 4°C in PBST containing primary antibodies. The following primary antibodies were used: monoclonal mouse anti‐orexin‐A (1:600, Santa Cruz Biotechnology, sc‐80,263) and polyclonal rabbit anti‐GABA (1:1000, Acris Antibodies 20,094). After staining with the primary antibodies, the sections were rinsed three times with PBST again and incubated with the secondary antibodies (AlexaFluor 488 donkey anti‐rabbit IgG, 1:1000, Jacksonimmuno, Inc.; AlexaFluor 488 goat anti‐mouse IgG, 1:1000, Abcam) for 2 h at room temperature (20–22°C). Next, after three washes with PBST, the brain slices were incubated with 4′,6‐diamidino‐2‐phenylindole (DAPI, 1:10000, Sigma‐Aldrich D9542) at room temperature for 10 min to stain the nuclei. Finally, after washing with PBST, the brain slices were placed on glass slides and covered with coverslips.

### 2.5 Imaging and data analysis

Whole‐brain sections were imaged under 10× or 20× magnification with the VS120 virtual microscopy slide scanning system (Olympus). A confocal microscope was used to obtain 20× or 40× magnified images of the brain sections to obtain more details (Olympus Fluoview 1000, Tokyo, Japan). For cell mapping of neurons, the ImageJ software was used to semi‐automatically quantify the neuronal bodies. Based on a map of the mouse brain, the boundaries of specific brain regions were depicted using ImageJ.^18^ We also used ImageJ to distinguish between the cells that co‐expressed DsRed and GFP for starter cell mapping. Then, we generated cell representation by applying the automatic wand (tracing) tool and bicubic interpolation to maximize neuronal fidelity. Next, we inverted the colorless regions to white and matched the contours of the cells to the corresponding brain regions based on the mouse brain atlas. Starter cells were binned at 0.12 mm along the anterior–posterior axis, with each coronal section image centered at the brain slice. For axonal varicosity counting, the images were captured using a 20× objective on the Olympus VS120 system. The axonal varicosity values of the whole brain were calculated semi‐automatically using the particle analyzing plugin in ImageJ. If the transverse diameters of the axons were >0.5 mm, the varicosities were defined.^19^ ImageJ was further used to outline the brain regions based on the reference brain atlas. The strength and direction of the linear relationship between subregions and cells or varicosity proportion were measured using the Pearson product–moment correlation coefficient.

## 3 RESULTS

### 3.1 Identification of monosynaptic inputs to LPAG glutamatergic neurons using an RV‐based system

**FIGURE 1:**
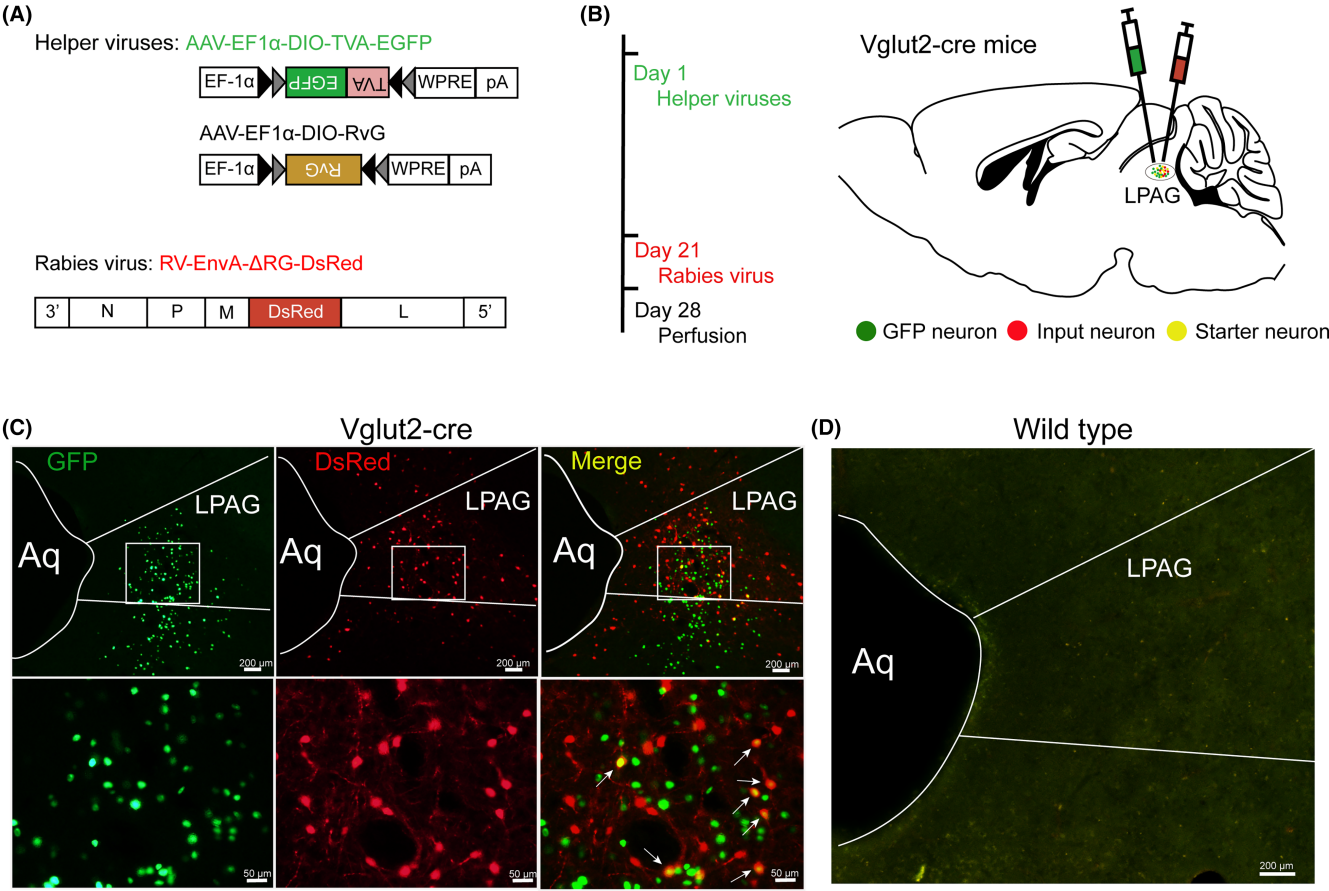
Experimental strategy for RV‐based retrograde tracing in LPAG glutamatergic neurons. (A) Design of viral vectors for RV‐based trans‐synaptic retrograde tracing, including helper viruses with Cre‐dependent expression of TVA receptor (AAV EF1α‐DIO‐TVA‐EGFP) and RvG (AAV‐EF1α‐DIO‐RvG). The RV was genetically modified by pseudotyping with EnvA (RV‐EnvA‐△RG‐DsRed). (B) Schematic of the injection procedure and experimental timeline for helper viruses and RV in the Vglut2‐Cre mouse. (C, D) Fluorescence images showing GFP‐ and DsRed‐expressing neurons in the LPAG after helper virus and RV injected in Vglut2‐Cre mouse and wild type mouse. The lower panels are the enlarged view of the white boxed region in the upper panels. Scale bar: 200 μm (upper panels and right panels), 50 μm (lower panels).

To identify monosynaptic inputs to the glutamatergic neurons in the LPAG, we used a cell type‐specific, RG‐deleted RV strategy^20^ in transgenic mice line expressing Cre recombinase in glutamatergic neurons (Vglut2‐Cre mice), which has been shown to label monosynaptic inputs of selected starter neurons with high specificity. On the first day, we injected two Cre‐dependent helper viruses (AAV‐EF1α‐DIO‐TVA‐EGFP and AAV‐EF1α‐DIO‐RvG) to express the enhanced green fluorescent protein (EGFP), avian‐specific retroviral receptor (TVA), and the rabies glycoprotein G (RG)^15^ in the unilateral LPAG in Vglut2‐Cre mice or wild‐type (WT) mice. Three weeks later, the modified RV (RV‐EnvA‐ΔRG‐DsRed) that only infects neurons expressing TVA, and requires RG to spread retrogradely to presynaptic neurons was injected into the same area in the same Vglut2‐Cre or WT mice. On the 28th day, the mice were perfused and their brains were processed (Figure 1A,B). To obtain a quantitative comparison between the brain samples, each sample was aligned with the Allen Mouse Brain Atlas after tissue sectioning and fluorescence imaging. After that, we manually identified and labeled the starter cells (expressing both DsRed and EGFP) and presynaptic neurons (expressing DsRed only), and registered their locations in the reference atlas. The starter cells were co‐infected with AAV helper viruses and RV, and they were restricted to the LPAG ipsilateral to the injection area in the Vglut2‐Cre mice (Figure 1C). The brains of the WT mice exhibited neither EGFP‐positive nor DsRed‐positive neurons in the LPAG (Figure 1D), showing that the expression of RG‐deleted RV strategy was highly cell‐type specific.

### 3.2 Whole‐brain input patterns to LPAG glutamatergic neurons

**FIGURE 2:**
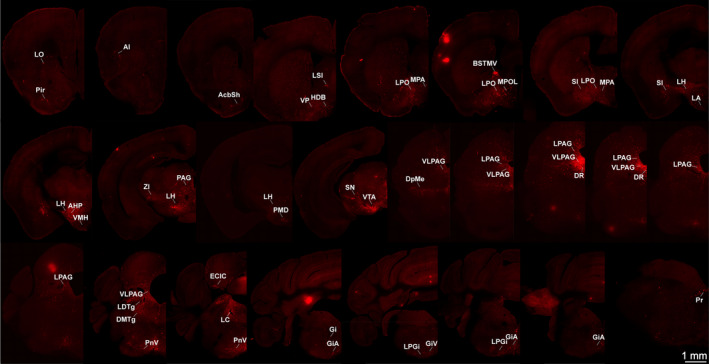
Representative images of monosynaptic inputs to LPAG glutamatergic neurons from the whole brain. Regions are labeled according to the mouse brain atlas. Scale bar: 1 mm. AI, agranular insular cortex; AcbSh, accumbens nucleus, shell; AHP, anterior hypothalamic area, posterior part; BSTL, bed nucleus of the stria terminalis, lateral division; CeMAD, central amygdaloid nucleus, medial division, anterodorsal part; DR, dorsal raphe nucleus; DMTg, dorsomedial tegmental area; ECIC, external cortex of the inferior colliculus; Gi, gigantocellular reticular nucleus; GiA, gigantocellular reticular nucleus, alpha part; GiV, gigantocellular reticular nucleus, ventral part; HDB, nucleus of the horizontal limb of the diagonal band; I, intercalated nuclei of the amygdala; LA, lateroanterior hypothalamic nucleus; LC, locus coeruleus; LPGi, lateral paragigantocellular nucleus; LPAG, lateral periaqueductal gray; LDTg, laterodorsal tegmental nucleus; LH, lateral hypothalamic area; LPO, lateral preoptic area; LSI, lateral septal nucleus, intermediate part; LO, lateral orbital cortex; MPA, medial preoptic area; MdD, medullary reticular nucleus, dorsal part; MHb, medial habenular nucleus; MPOL, medial preoptic nucleus, lateral part; Pir, piriform cortex; PAG, periaqueductal gray; PnV, pontine reticular nucleus, ventral part; Pr, prepositus nucleus; PMD, premammillary nucleus, dorsal part; SI, substantia innominata; SN, substantia nigra; TC, tuber cinereum area; VP, ventral pallidum; VMH, ventromedial hypothalamic nucleus; VTA, ventral tegmental area; VLPAG, ventrolateral periaqueductal gray.

**FIGURE 3:**
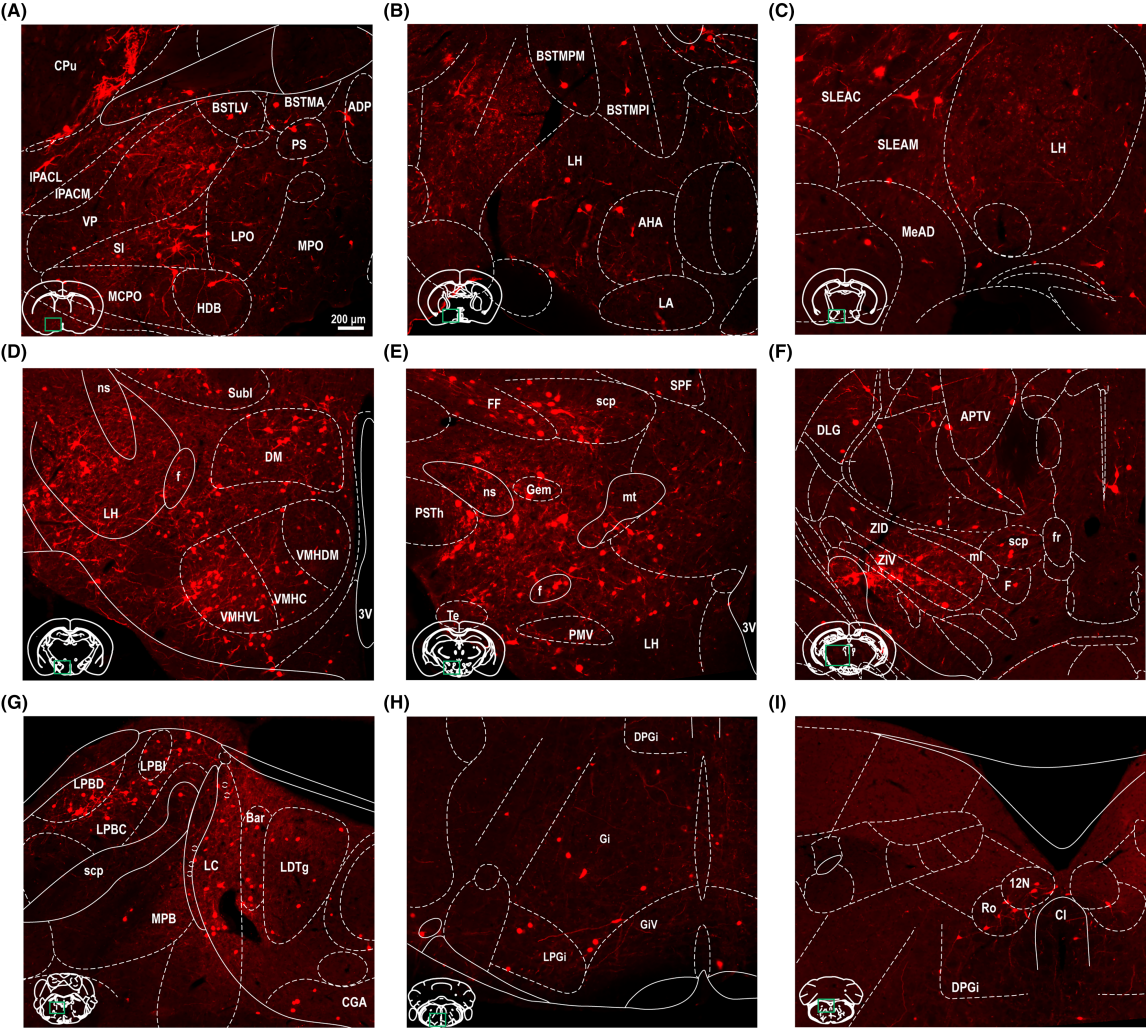
Schematic representation of the typical regions with monosynaptic inputs to LPAG glutamatergic neurons. (A–I) Primary inputs originated from regions involved in physiological behaviors (e.g., VP, LH, MPB, LC, LDT, DPGi, LPGi, and Giv). Scale bar: 200 μm. 12N, hypoglossal nucleus; 3V, 3rd ventricle; ADP, anterodorsal preoptic nucleus; AHA, anterior hypothalamic area, anterior part; APTV, anterior pretectal nucleus, ventral part; Bar, Barrington's nucleus; BSTLV, bed nucleus of the stria terminalis, lateral division, ventral part; BSTMA, bed nucleus of the stria terminalis, medial division, anterior part; BSTMPI, bed nucleus of the stria terminalis, medial division, posterointermediate part; BSTMPM, bed nucleus of the stria terminalis, medial division, posteromedial part; CGA, central gray, alpha part; CI, caudal interstitial nucleus of the medial longitudinal fasciculus; CPu, caudate putamen; DLG, dorsal lateral geniculate nucleus; DM, dorsomedial hypothalamic nucleus; DPGi, dorsal paragigantocellular nucleus; f, fornix; F, nucleus of the fields of Forel; FF, fields of Forel; fr, fasciculus retroflexus; Gem, gemini hypothalamic nucleus; Gi, gigantocellular reticular nucleus; GiV, gigantocellular reticular nucleus, ventral part; HDB, nucleus of the horizontal limb of the diagonal band; IPACL, interstitial nucleus of the posterior limb of the anterior commissure, lateral part; IPACM, interstitial nucleus of the posterior limb of the anterior commissure, medial part; LA, lateroanterior hypothalamic nucleus; LC, locus coeruleus; LDTg, laterodorsal tegmental nucleus; LH, lateral hypothalamic area; LPBC, lateral parabrachial nucleus, central part; LPBD, lateral parabrachial nucleus, dorsal part; LPBI, lateral parabrachial nucleus, internal part; LPGi, lateral paragigantocellular nucleus; LPO, lateral preoptic area; MCPO, magnocellular preoptic nucleus; MeAD, medial amygdaloid nucleus, anterodorsal; ml, medial lemniscus; MPO, medial preoptic nucleus; mt, mamillothalamic tract; ns, nigrostriatal bundle; PMV, premamillary nucleus, ventral part; PS, parastrial nucleus; PSTh, parasubthalamic nucleus; Ro, nucleus of Roller; scp, superior cerebellar peduncle; SI, substantia innominata; SLEAC, sublenticular extended amygdala, central part; SLEAM, sublenticular extended amygdala, medial part; SPF, subparafascicular thalamic nucleus; Subl, subincertal nucleus; Te, terete hypothalamic nucleus; VMHC, ventromedial hypothalamic nucleus, central part; VMHDM, ventromedial hypothalamic nucleus, dorsomedial part; VMHVL, ventromedial hypothalamic nucleus, ventrolateral part; VP, ventral pallidum; ZID, zona incerta, dorsal part; ZIV, zona incerta, ventral part.

To determine the inputs to LPAG glutamatergic neurons, the brains of these injected mice were cut serially in coronal sections after adequate infection time (Figure 2). Sections from a representative Vglut2‐Cre mouse (Figure 2) revealed that DsRed‐labeled presynaptic neurons were observed in many brain nuclei. DsRed‐labeled afferent neurons were largely located in the hypothalamus, while some were also found in the thalamus, midbrain, and medulla. Moreover, a few input neurons were found in the cortex, superior colliculus and pons (Figure 2). Next, to display the LPAG presynaptic neurons in greater detail (Figure 3), representative images of inputs from typical subregions were enlarged and selected, such as the lateral hypothalamic area (LH), lateral preoptic area (LPO), substantia innominata (SI), ventral pallidum (VP), lateral globus pallidus (LGP), medial preoptic area (MPA), posterior hypothalamic area (PH), bed nucleus of the stria terminalis, lateral division, intermediate part (BSTLI), zona incerta (ZI), dorsomedial hypothalamic nucleus (DM), medial preoptic nucleus, medial part (MPOM), intermediate reticular nucleus (IRt), subincertal nucleus (Subl), caudate putamen (CPu), and gigantocellular reticular nucleus (Gi).

### 3.3 Immunofluorescence of DsRed‐labeled neurons and several markers of physiological behaviors

**FIGURE 4:**
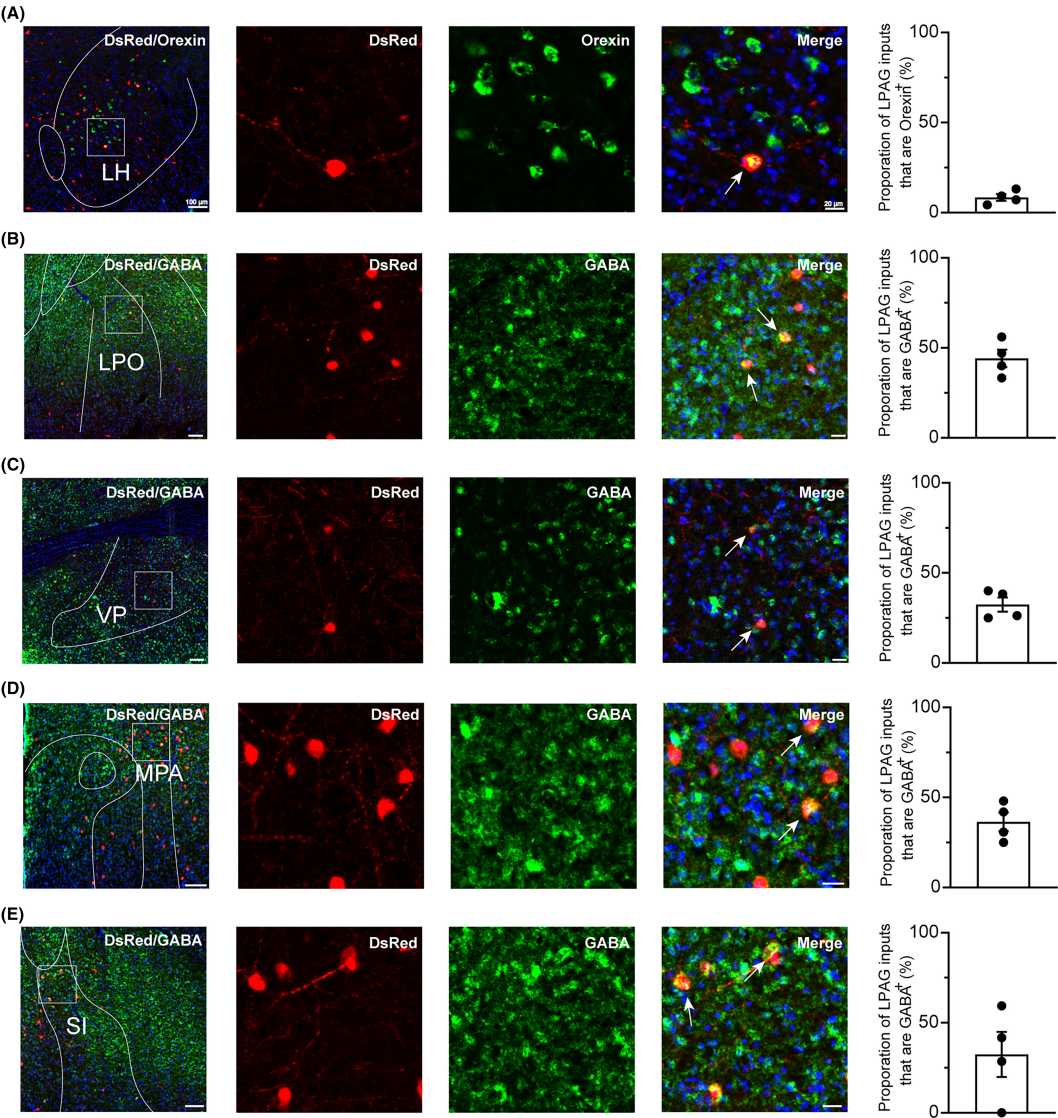
Immunofluorescence of DsRed and several markers of physiological behaviors. (A–E) Images showing DsRed‐labeled afferent neurons colocalized with several markers of physiological behavior regulation in typical brain regions. Enlarged views of the white boxed regions in the left‐most images are shown in the three right images. Colocalized neurons are indicated by arrows in the rightmost images. Images showing that some DsRed‐labeled neurons were colocalized with orexin^+^ neurons in the LH, GABAergic neurons in the LPO, VP, MPA, or SI. The quantification of DsRed^+^ cells that are positive for special biomarkers is presented in the rightmost columns. *n* = 4, each data point represents one experimental animal. Scale bar: 100 μm (left‐most images), 20 μm (three right images). GABA, γ‐aminobutyric acid; LH, lateral hypothalamic area; LPO, lateral preoptic area; MPA, medial preoptic area; SI, substantia innominate; VP, ventral pallidum.

Next, we determined the colocalization of inputs to the LPAG glutamatergic neurons using several neuronal markers associated with important physiological functions via immunofluorescence. In the LH, the inputs to LPAG glutamatergic neurons were found to be partly colocalized with orexin (also known as hypocretin) (8.41% ± 1.86% in Figure 4A), which is a central hub for the integration of a wide range of inputs from the brain regions that regulate physiological homeostasis and complex behaviors.^21^ The LH orexin neurons are wake‐promoting neurons.^22^, ^23^, ^24^ Deficiency of orexin or its receptor leads to narcolepsy in animals.^25^ In addition, the LH orexin system is also involved in appetitive, stress response, and other behaviors necessary for survival.^26^, ^27^ The dense monosynaptic projections in the LH to the LPAG glutamatergic neurons may be to excitatory glutamatergic or inhibitory GABAergic neurons, because a large number of cell populations in the LH are glutamatergic and GABAergic neurons.^28^ The input neurons from the LPO to LPAG glutamatergic neurons were mostly colocalized with GABA neurons (44.10% ± 4.86% in Figure 4B), where a center for the induction of NREM and REM sleep is located.^29^ In the LPO, GABAergic cell populations are vital for arousal and sleep homeostasis. Optogenetic stimulation of GABAergic neurons in the LPO of mice during sleep leads to rapid wake induction, and the awake state produced is characterized by increased EEG theta activity. These findings suggest a role of LPO GABAergic neurons in linking arousal to sleep homeostasis.^30^ In addition, VP is mainly composed of GABAergic neurons, and recent studies have found that VP GABAergic neurons are essential for the control of reward‐seeking behaviors, approach responses, and wakefulness associated with motivation.^31^, ^32^, ^33^ Notably, in our study, a large number (32.41% ± 3.92%) of dsRed‐labeled neurons in the VP were GABAergic populations (Figure 4C). In addition, we also found that 36.45% of the input neurons from the MPA and 41.67% from the SI to LPAG glutamatergic neurons were also GABAergic (Figure 4D,E). These brain nuclei are involved in several physiological behaviors, such as hunting, anxiety, and sleep–wake regulation.^34^, ^35^, ^36^, ^37^, ^38^

### 3.4 Analysis of input neurons innervating LPAG glutamatergic neurons

**FIGURE 5:**
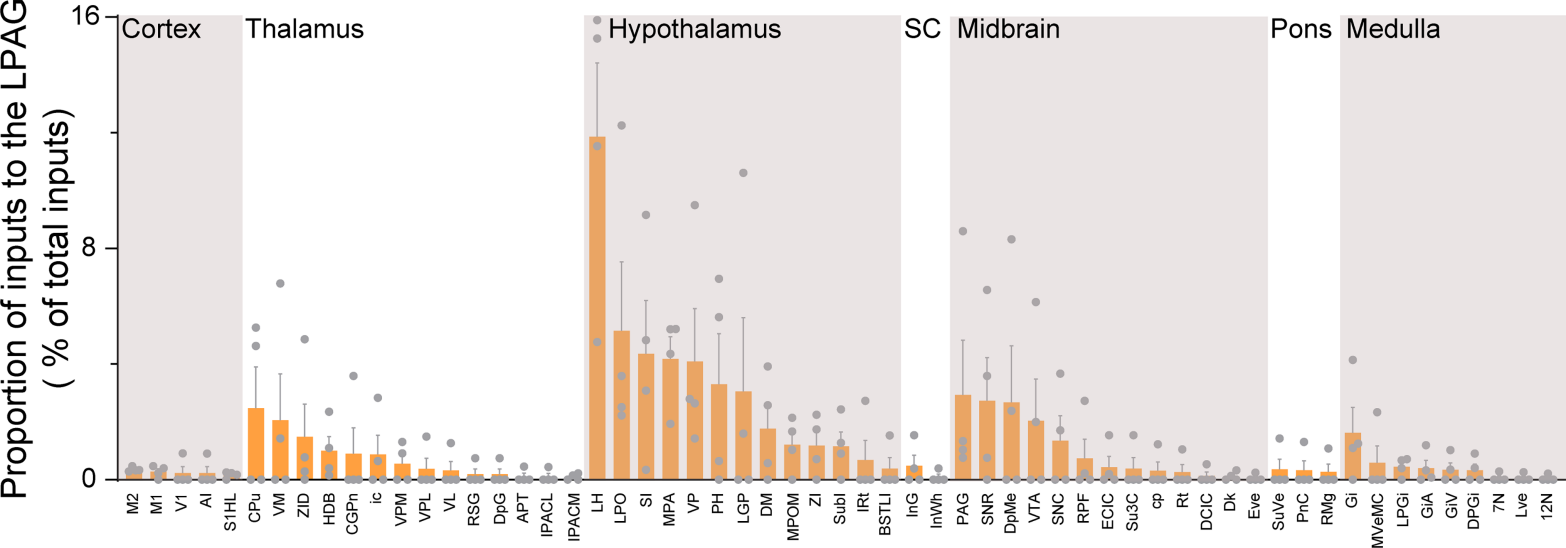
Statistical analysis of the whole‐brain distribution of monosynaptic inputs to LPAG glutamatergic neurons. Average proportion of DsRed‐labeled neurons in brain regions with more than 0.05% average input proportions from LPAG glutamatergic neurons. Error bars represent the standard error of mean. *n* = 4, each data point represents one experimental animal. Brain regions are grouped into seven general structures listed at the top, and specific brain regions are listed at the bottom. AI, agranular insular cortex; APT, anterior pretectal nucleus; BSTLI, bed nucleus of the stria terminalis, lateral division, intermediate part; CGPn, central gray of the pons; cp, cerebral peduncle, basal part; CPu, caudate putamen; DCIC, dorsal cortex of the inferior colliculus; Dk, nucleus of Darkschewitsch; DM, dorsomedial hypothalamic nucleus; DpG, deep gray layer of the superior colliculus; DPGi, dorsal paragigantocellular nucleus; DpMe, deep mesencephalic nucleus; ECIC, external cortex of the inferior colliculus; Eve, nucleus of origin of efferents of the vestibular nerve; Gi, gigantocellular reticular nucleus; GiA, gigantocellular reticular nucleus, alpha part; GiV, gigantocellular reticular nucleus, ventral part; HDB, nucleus of the horizontal limb of the diagonal band; ic, internal capsule; InG, intermediate gray layer of the superior colliculus; InWh, intermediate white layer of the superior colliculus; IPACL, interstitial nucleus of the posterior limb of the anterior commissure, lateral part; IPACM, interstitial nucleus of the posterior limb of the anterior commissure, medial part; IRt, intermediate reticular nucleus; LGP, lateral globus pallidus; LH, lateral hypothalamic area; LPGi, lateral paragigantocellular nucleus; LPO, lateral preoptic area; Lve, lateral vestibular nucleus; M1, primary motor cortex; M2, secondary motor cortex; MPA, medial preoptic area; MPOM, medial preoptic nucleus, medial part; MVeMC, medial vestibular nucleus, magnocellular part; PAG, periaqueductal gray; PH, posterior hypothalamic area; PnC, pontine reticular nucleus, caudal part; RMg, raphe magnus nucleus; RPF, retroparafascicular nucleus; RSG, retrosplenial granular cortex; Rt, reticular thalamic nucleus; S1HL, primary somatosensory cortex, hindlimb region; SC, superior colliculus; SI, substantia innominata; SNC, substantia nigra, compact part; SNR, substantia nigra, reticular part; Su3C, supraoculomotor cap; SubI, subincertal nucleus; SuVe, superior vestibular nucleus; V1, primary visual cortex; VL, ventrolateral thalamic nucleus; VM, ventromedial thalamic nucleus; VP, ventral pallidum; VPL, ventral posterolateral thalamic nucleus; VPM, ventral posteromedial thalamic nucleus; VTA, ventral tegmental area; ZI, zona incerta; ZID, zona incerta, dorsal part; 12N, hypoglossal nucleus; 7N, facial nucleus.

Next, in order to better identify the distribution of these DsRed‐labeled neurons, we performed statistical analysis after identifying the brain regions with monosynaptic inputs to LPAG glutamatergic neurons based on standard mouse brain atlases. We divided the whole brain into seven brain structures, namely the cortex, thalamus, hypothalamus, superior colliculus, midbrain, pons, and medulla. Then, we calculated the proportion of DsRed‐labeled afferent neurons of each brain region in the whole brain. We defined the brain regions in which DSRed‐labeled neurons accounted for >0.05% of the total labeled neurons as the nuclei with monosynaptic connections with LPAG glutamatergic neurons and generated a list of whole‐brain inputs to LPAG glutamatergic neurons (Figure 5). We found that the total afferent neurons, including 59 nuclei, originated in the seven brain structures: the cortex, thalamus, hypothalamus, superior colliculus, midbrain, pons, and medulla (*n* = 4 mice). We found that the highest numbers of inputs to LPAG were localized in the hypothalamus. The anatomical and functional connectivity of the hypothalamic‐PAG circuit, especially the ventromedial hypothalamus and ventrolateral area (VMHvl), to the LPAG has been reported previously,^10^ which is highly consistent with our findings. We also found that the top seven nuclei, the LH (11.86% ± 2.55%), LPO (5.14% ± 2.39%), SI (4.35% ± 1.85%), VP (4.09% ± 1.83%), PH (3.30% ± 1.75%), LGP (3.05% ± 2.55%), and MPA (4.18% ± 0.77%), that projected most densely to the LPAG glutamatergic neurons were located in the hypothalamus. In addition, the CPu (2.47% ± 1.43%) and the VM (2.05% ± 1.61%) in the thalamus, the PAG (2.93% ± 1.89%), SNR (2.73% ± 1.49%), DpMe (2.67% ± 1.96%) and VTA (2.04% ± 1.45%) in the midbrain also had strong projections to LPAG glutamatergic neurons. Beyond that, in the thalamus, hypothalamus, midbrain, and medulla, multiple nuclei had monosynaptic connections with proportions >1% of the total input neurons to LPAG glutamatergic neurons, including the ZID (1.48% ± 1.14%), ZI (1.17% ± 0.51%), DM (1.77% ± 0.90%), MPOM (1.22% ± 0.46%), IRt (1.42% ± 0.24%), SubI (1.15% ± 0.50%), SNC (1.34% ± 0.87%) and Gi (1.62% ± 0.88%).

**FIGURE 6:**
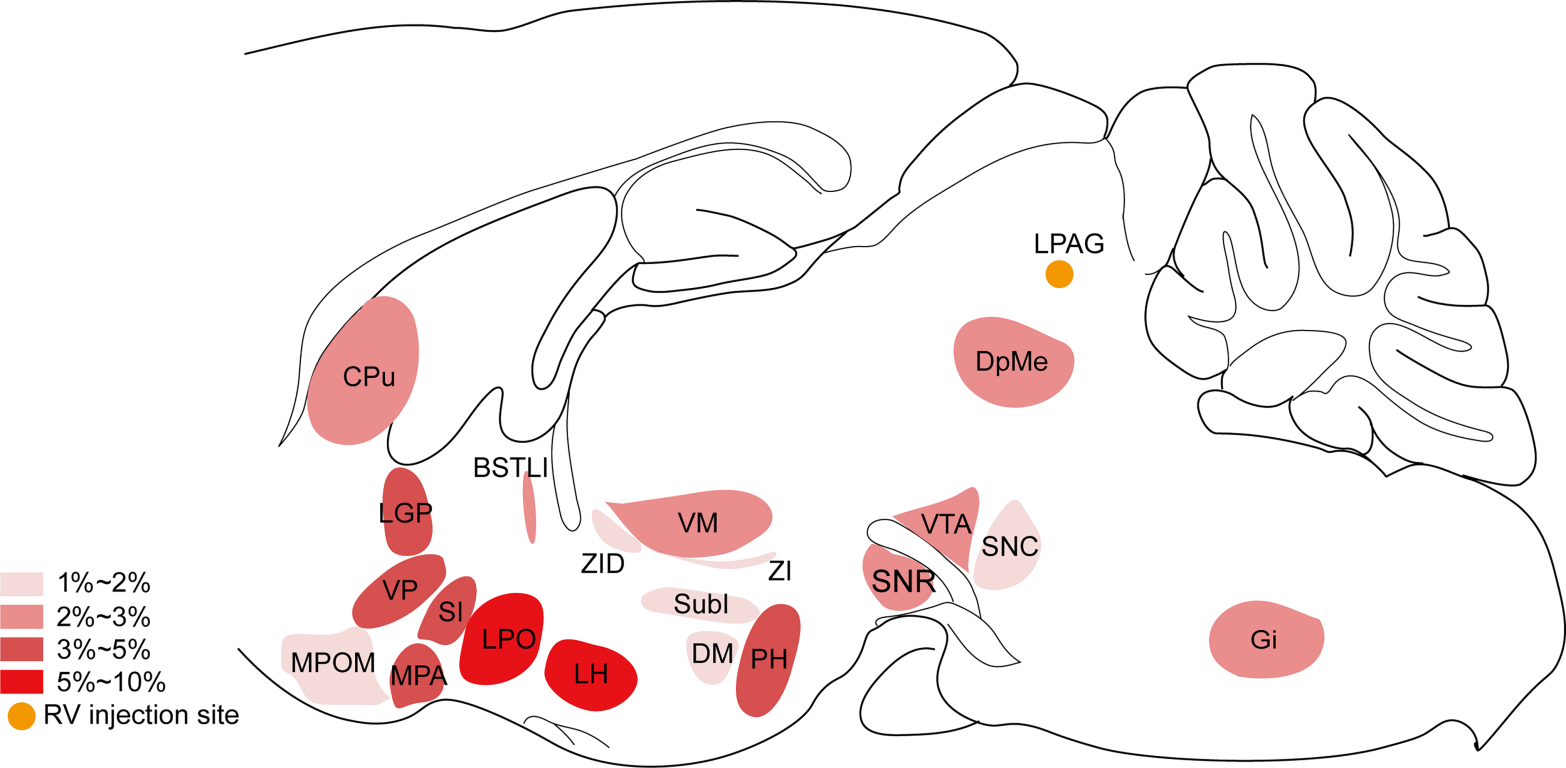
Schematic diagrams showing the distribution of the monosynaptic inputs innervating the LPAG glutamatergic neurons. Sagittal sections for a schematic illustration of whole‐brain inputs to LPAG glutamatergic neurons in Vglut2‐Cre mice. BSTLI, bed nucleus of the stria terminalis, lateral division, intermediate part; CPu, caudate putamen; DM, dorsomedial hypothalamic nucleus; DpMe, deep mesencephalic nucleus; Gi, gigantocellular reticular nucleus; LGP, lateral globus pallidus; LH, lateral hypothalamic area; LPAG, lateral periaqueductal gray; LPO, lateral preoptic area; MPA, medial preoptic area; MPOM, medial preoptic nucleus, medial part; PH, posterior hypothalamic area; SI, substantia innominata; SNC, substantia nigra, compact part; SNR, substantia nigra, reticular part; SubI, subincertal nucleus; VM, ventromedial thalamic nucleus; VP, ventral pallidum; VTA, ventral tegmental area; ZI, zona incerta; ZID, zona incerta, dorsal part.

Finally, to compare the broad distribution of the input neurons more intuitively in the whole brain, we showed the sagittal sections for schematic illustrations of the proportion of input neurons within each nucleus of the whole brain monosynaptic inputs to LPAG glutamatergic neurons, which clearly showed that the numerous afferent neurons of the total whole brain were located in the hypothalamus (Figure 6).

However, a technical limitation of our study is that the olfactory bulb was often damaged when the mice brain was cut to create coronal sections, which led to a significant underestimation of labeling in the olfactory bulb. In conclusion, our results provide insights into input distribution to LPAG glutamatergic neurons in the whole brain, except for the olfactory bulb.

## 4 DISCUSSION

The LPAG is involved in the regulation of a variety of brain functions, including defensive, offensive,^1^ and social behaviors^2^, ^7^ and pain.^8^ In addition, our recent study revealed an important role of LPAG glutamatergic neurons in the sleep–wake neural circuit. It is crucial to explore the afferent inputs to LPAG glutamatergic neurons for understanding their regulation in brain functions. In this study, we used a cell type‐specific, RG‐deleted RV strategy and accurately determined the whole‐brain monosynaptic inputs to glutamatergic neurons in the LPAG. We found that LPAG glutamatergic neurons received extensive direct inputs from the whole brain. Moreover, these input neurons preferentially originated from a wide range of nuclei in the hypothalamus, such as the LH, LPO, SI, VP, LGP, and MPA. Taken together, our results revealed a comprehensive map of the presynaptic patterns that may control LPAG glutamatergic neuron activity, which contributes to a better understanding of the role of LPAG in multiple functions and behaviors.

### 4.1 Advantages of specific trans‐synaptic tracing compared to traditional retrograde tracing

In recent years, the neural connectivity of the LPAG has been investigated because it has pivotal roles in multiple brain functions. Most previous studies on the inputs to the LPAG have focused on specific regions connected to the LPAG, for example, the auditory cortex,^11^ anterior cingulate cortex,^39^ lateral parabrachial nucleus,^40^ LH, CeA, and ZI.^12^ These findings provide some evidence for brain‐wide neuronal inputs to the LPAG. In addition, previous investigations used conventional tracing techniques, such as cholera‐toxin subunit B (CTB) and wheat germ agglutinin conjugated horseradish peroxidase (WGA‐HRP). Keay et al.^41^ injected the retrograde tracer CTB into the LPAG of rats and revealed the spinal afferents to the LPAG. The reciprocal connections between the medial preoptic area and the midbrain PAG in rats were also found via exploiting the WGA‐HRP.^42^ These studies had several limitations because the input of specific brain regions to the LPAG is difficult to assess based on whole‐brain mapping. Traditional retrograde tracers, such as CTB and HRP, cannot identify the inputs to the LPAG‐specific cell types (Table 1).

**TABLE 1:** The differences between RV‐based trans‐synaptic tracing and previous tracing studies on the LPAG.

Retrograde tracer	Animal	Neuron type	Key results	Ref.
RV‐based retrograde tracers	Vglut2‐Cre mouse	Glutamatergic neurons	Investigated whole‐brain monosynaptic inputs to the LPAG glutamatergic neurons	
CTB	Rat	All neurons	Verified the spinal input to the LPAG arose predominantly from neurons in the upper cervical (C1–4) and sacral spinal cord	^41^
WGA‐HRP	Rat	All neurons	Verified the reciprocal connections between the PAG and medial preoptic area	^42^
WGA‐HRP	Cat	All neurons	Verified the upper cervical spinal cord input to distinct regions of the PAG	^70^

In this study, we used a RV‐mediated retrograde tracing system in Vglut2‐Cre mice, which allowed specific labeling of whole‐brain monosynaptic inputs to the LPAG glutamatergic neurons (Table 1). We found that the majority of these inputs originate from the LH, LPO, and VP of the hypothalamus, which is also consistent with previous findings.^43^ In addition, the SI, LGP, and MPA of the hypothalamus contained a large number of DsRed‐labeled cells. Moreover, relatively dense inputs to the LPAG also originated from the CPu in the thalamus and the Gi in the medulla. In conclusion, our findings provide a comprehensive map of the presynaptic patterns that control LPAG glutamatergic neurons.

### 4.2 Implications of LPAG activity in offensive and defensive behaviors

Studies conducted in recent decades have firmly established the pivotal role of LPAG in the defensive and offensive neural circuit. LPAG receives the densest projections from the VMHvl,^43^ which is a critical hub for the attack behavior.^44^ Excitation of the LPAG can induce defensive behaviors characterized by alertness, freezing, and escape.^45^ The different neural populations of LPAG may play different roles in offensive and defensive behaviors. LPAG GABAergic neurons are required for prey detection, chase, and attack, while LPAG glutamatergic neurons are selectively required for attack.^12^ In addition, LPAG glutamatergic neurons, rather than GABAergic neurons, receive direct input from auditory cortical centers and mediate sound‐driven defensive behavior.^11^ A recent study revealed the importance of the hypothalamic‐midbrain circuit in coordinating aggressive action. The LPAG glutamatergic cells receive preferential projection from the VMHvl glutamatergic cells, and chemogenetic inactivation of the LPAG glutamatergic neurons results in aggression‐specific deficits.^10^ We mapped the monosynaptic inputs to the LPAG glutamatergic cells and found that the projections from the LPAG glutamatergic populations were mainly distributed in the hypothalamus. Furthermore, the top nuclei projections to the LPAG were also involved in offensive and defensive behaviors, for example, the LH^46^ and SI,^47^ which suggests that LPAG may play an important role in defensive‐offensive circuits by integrating complex signals from the hypothalamus.^10^

### 4.3 Neuroanatomical evidence for the potential role of the LPAG in sleep–wake regulation

The functional columns of the PAG play important roles in the sleep–wake cycle. Zhong et al.^48^ showed that NTS‐expressing glutamatergic neurons in the VLPAG are preferentially active during NREM sleep, and their activation strongly promotes NREM sleep. The activation of GABAergic neurons in the VLPAG suppresses the initiation and maintenance of REM sleep and consolidates NREM sleep.^49^ Activation of neurotensinergic neurons in the LPAG could promote NREM sleep.^13^

In this study, we found that the LPAG glutamatergic populations receive strong inputs from several brain regions associated with sleep–wake regulation. MCH‐expressing neurons and orexin/hypocretin neurons in the LH are important for sleep–wake regulation.^22^, ^50^ The LH GABAergic and glutamatergic neurons also mediate the sleep–wake cycle.^51^, ^52^ Our results revealed that LPAG glutamatergic neurons received the densest projections from the LH; thus, the LH‐LPAG neural circuit may be important for sleep–wake regulation. Furthermore, we also observed that the LPO, VP, SI, LGP, and MPA sent abundant projections to the LPAG glutamatergic neurons; more importantly, these nuclei are involved in sleep–wake regulation.^29^, ^30^, ^32^, ^36^, ^37^, ^53^, ^54^, ^55^ The direct inputs from several sleep–wake related nuclei located in the hypothalamus to the LPAG glutamatergic neurons suggested that the LPAG may function via integrating sleep–wake regulatory signals from the hypothalamus.

### 4.4 Neural circuitry underlying modulation of LPAG in pain responses

The LPAG plays a vital role in pain‐related behavioral responses. Several clinical studies have shown that the functional connectivity of LPAG is disrupted in patients with pain.^56^ In addition, LPAG mediates pain avoidance behaviors. For example, increased signals from the anterior cingulate cortex to the DL/LPAG are critical for fear avoidance in chronic pain disability.^39^ The hypothalamus, especially the LH, is also involved in pain perception and promotion.^57^, ^58^ The lateral septum‐LH circuit is critical for pain modulation,^57^ and activation of the orexin system facilitates pain control.^59^ In addition, Siemian et al.^60^ demonstrated that the LH parvalbumin‐positive (LH^PV^) glutamatergic neurons have a potential role as a target for analgesia, in which the LH^PV^ neurons‐VLPAG axonal projections play a vital role. More interestingly, an increasing number of studies have suggested bidirectional regulation of sleep–wake and pain behavior.^61^, ^62^ In our research, we found that numerous hypothalamic subregions associated with sleep–wake regulation, especially LH, send dense projections to the LPAG, which may provide several new directions for future research. For example, future studies should explore whether the LPAG can achieve bidirectional control of sleep–wake regulation and pain processing and whether this process is related to the integration of signals from the hypothalamus.

### 4.5 The functional implications of LPAG activity for heart rate and respiration

LPAG is involved in fear, defensive, sleep–wake, pain and other behavioral functions. These behaviors are accompanied by changes in heart rate (HR) and respiration, which demonstrates the significant potential implications of LPAG activity for HR and respiratory control. For HR, manipulating the activity of LPAG neurons can effectively alter cardiovascular effects, including bradycardia or increases in HR.^63^, ^64^, ^65^, ^66^ The different effects may result from the differential activation of excitatory or inhibitory neurons in the LPAG. In addition, the cardiovascular response controlled by hypothalamic neurons is largely dependent on the activity of PAG neurons,^63^, ^64^, ^65^, ^66^ demonstrating the vital role of the hypothalamus–PAG circuit in HR control. For respiration, the PAG‐substructures of different animals play differential roles in respiration control.^67^ For example, in humans, the VLPAG is involved in anticipation of breathing difficulty, whereas difficult breathing is associated with activity in the LPAG.^68^ In addition, Subramanian et al.^69^ stimulated the LPAG in cats and found three types of respiratory responses, including tachypnea, inspiratory apneusis, and respiratory changes in the context of vocalization. In brief, as a control center for behavioral regulation, the PAG may be involved in the integration of sensory signals from the periphery, including HR and respiration.

In conclusion, we mapped the monosynaptic afferents to the LPAG glutamatergic neurons and found that they received projections from other brain areas, especially the nuclei localized in the hypothalamus. This suggests a vital role of LPAG glutamatergic neurons in a wide range of physiological and pathological functions, including sleep–wake regulation, offensive‐defensive behaviors, and pain response. We also summarized the involved physiological behaviors of nuclei that input to LPAG glutamatergic neurons (Table 2), which may help us better understand the implications of LPAG in a variety of physiological and behavioral functions. Therefore, our neuroanatomical data could be useful for future explorations of the LPAG and provide a structural framework for the underlying neural mechanisms related to certain physiological functions.

**TABLE 2:** The proportion (>2%) and involved physiological behaviors of nuclei that input to the LPAG glutamatergic neurons.

Brain region	Nucleus	Proportion	Physiological behaviors	Ref.
Thalamus	CPu	2.47%	cognition; goal‐directed action; habit formation	71, 72
	VM	2.05%	pain; goal‐directed action; sleep–wake behaviors; attention	73, 74, 75
Hypothalamus	LH	11.86%	sleep–wake behaviors; appetitive behaviors; re‐stress response; offensive/defensive behaviors; pain	22, 26, 27, 46, 60
	LPO	5.14%	sleep–wake behaviors; reward	30, 76
	SI	4.35%	sleep–wake behaviors; depression; aversive behaviors	37, 77, 78
	VP	4.09%	motivation; depression; reward‐seeking behaviors; sleep–wake behaviors; approach responses; feeding behaviors	31, 32, 33
	LGP	3.05%	locomotion; REM sleep; defensive behaviors	36, 79
	MPA	4.18%	hunting; anxiety; torpor; sleep–wake behaviors	34, 35, 38, 80
	PH	3.30%	cognition; memory; anxiety; locomotion; sleep–wake behaviors	81, 82, 83, 84
Midbrain	SNR	2.73%	pain; memory; sleep–wake behaviors; locomotion	85, 86, 87
	PAG	2.93%	pain; offensive/defensive behaviors; social; antinociceptive; itch‐scratching; emotion; sleep–wake behaviors; HR; respiration	1, 2, 3, 4, 5, 13, 66, 69, 88
	DpMe	2.67%	REM sleep; licking behaviors	89, 90
	VTA	2.04%	reward; depression; sleep–wake behaviors; motivation; memory; aversion; learning	32, 91

## 5 CONCLUSIONS

Our study confirmed that the LPAG glutamatergic neurons received dense projections from the hypothalamus, and dsRed‐labeled neurons were colocalized with several markers of physiological behaviors. The pivotal role of glutamatergic neurons in the physiological behaviors regulated by the LPAG was further confirmed based on neuroanatomical terms.

## AUTHOR CONTRIBUTIONS

YQW and ZLH contributed to the study concept and design and drafting/revising of the manuscript for content. WXM and LL contributed to running of the experiments, acquisition of the data, and figure drawing. LXK, HZ, and PCY contributed to acquisition of the data and statistical analysis. All authors have read and agreed to the published version of the manuscript.

## CONFLICT OF INTEREST STATEMENT

All authors declare no conflict of interest.

## Data Availability

The data that support the findings of this study are available on request from the corresponding author. The data are not publicly available due to privacy or ethical restrictions.
